# Aquifer Microbial Communities Differentially Display Metabolisms Capable of Secondary Effects on Uranium Speciation Across a Former Metal Processing Site

**DOI:** 10.1111/1462-2920.70411

**Published:** 2026-09-08

**Authors:** Catherine J. Pettinger, Allondra M. Woods, Raymond H. Johnson, Charles J. Paradis, Erica L.‐W. Majumder

**Affiliations:** ^1^ Department of Bacteriology University of Wisconsin‐Madison Madison Wisconsin USA; ^2^ Biological Engineering Department Utah State University Logan Utah USA; ^3^ Contractor to the U.S. Department of Energy Office of Legacy Management RSI EnTech, LLC Grand Junction Colorado USA; ^4^ Department of Geosciences University of Wisconsin at Milwaukee Milwaukee Wisconsin USA

**Keywords:** aquifer communities, microbiology metabolism, reduction–oxidation reactions, subsurface microbiology, uranium biogeochemistry

## Abstract

Groundwater contamination presents worldwide challenges, yet remediation solutions in variably oxidized regions are limited, with co‐interactions between contaminant metals and microbial reactions occurring. Here we present a genomic and metabolic study into the biogeochemistry of a uranium‐contaminated surficial aquifer site in Riverton, WY. We identified unique communities that varied based on geochemistry, geography, and compartment, matching other microbial subsurface studies. Cross‐site metabolism tests showed communities had functional capabilities of nitrogen respiration, manganese reduction, iron reduction, and sulfide oxidation. None of the sites displayed evidence of direct U‐bioreduction nor ammonium oxidation. Only former tailings area sites nearest a retention pond and a downgradient oxbow lake exhibited sulfate reduction metabolisms. This was contrary to our hypothesis of near‐river downgradient groundwater sites having U and S reduction capability. Most communities which showed S reduction capacity exhibited Fe oxidation capacity. Modelling demonstrated U as calcium uranyl carbonates. Based on our metabolism tests and known mineral and microbial metabolism reduction potentials, this suggests U reduction could be achieved via a secondary reaction with biogenically‐formed sulfide. Of 11 sites tested, this is possible in four. Microbial metabolisms were analysed for capacity to influence U reduction–oxidation and provide microbial context to site remediation enhanced flushing efforts.

## Graphical abstract

We performed microbial membership and metabolism measurements across a uranium‐contaminated site's surface and ground waters, then performed analyses relating these metrics to geochemistry at the site. Findings showed variations in the membership, yet mainly similar functional capabilities. Metabolic differences were explained in relation to uranium cycling and remediation implications.

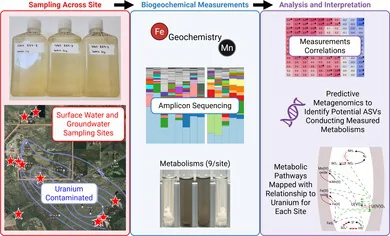

## 1 Introduction

Groundwater contamination at uranium mill sites presents a biogeochemical challenge across the United States and world. Access to freshwater is an issue recognized globally by World Health Organization (2025) and United Nations Environment Programme (2024), and more regionally in the Fifth National Climate Assessment which predicted water shortages to much of the United States, including the Western regions of the USA, over the next 15 years (Payton et al. 2023). The closure of World War II and Cold War era U mining, processing, and testing sites led to hazardous wastes at over 150 locations which still persist and require continual monitoring (U.S. Department of Energy 2025).

**FIGURE 1:**
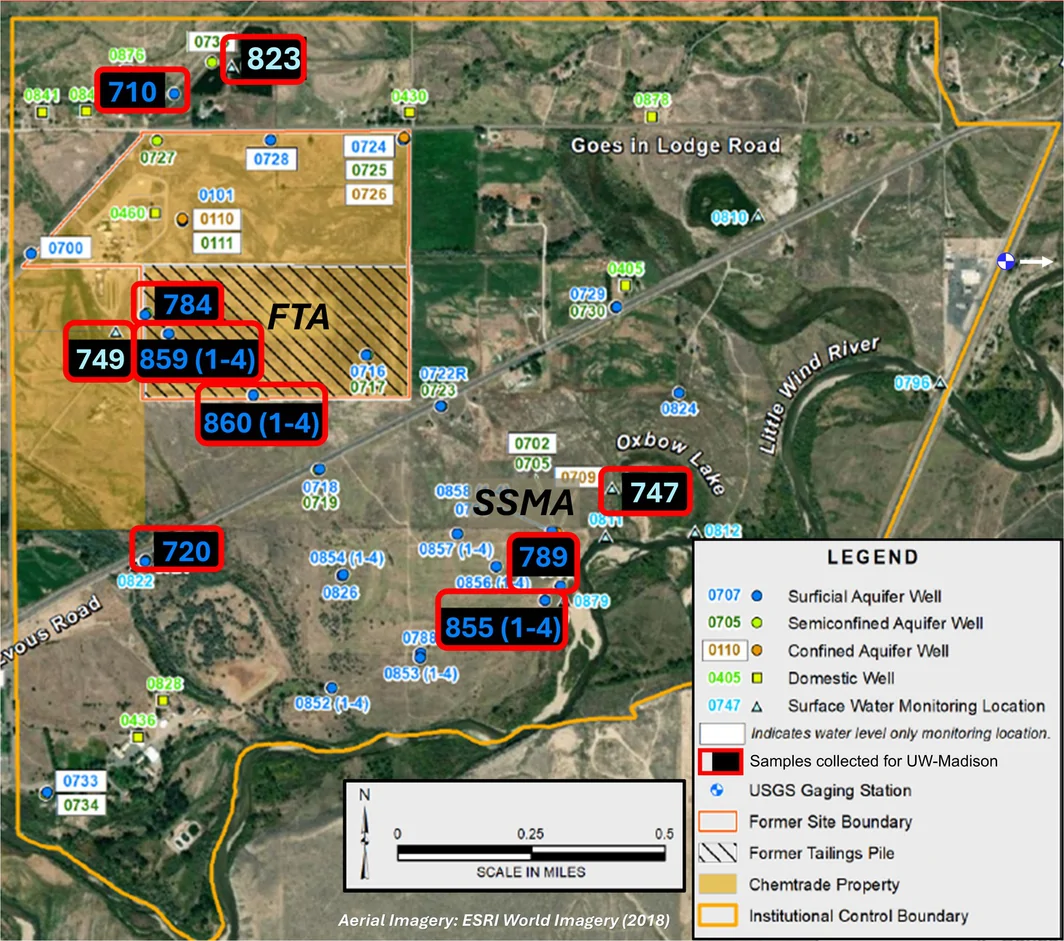
A sampling map of the Riverton site adapted from 2023 Verification Monitoring Report (U.S. Department of Energy 2024). The 11 sampling sites are indicated with a red box surrounding a blue number. Samples were collected from multi‐channel wells 855, 859, and 860 and surficial aquifer wells 710, 720, 784, and 789. General groundwater flow is from the Former Tailings Area (FTA) area towards the Little Wind River, with site 710 described as “upgradient”, sites 784, 859, and 860 within the FTA, and sites 789 and 855 downgradient at the St. Stephens Mission Area (SSMA). Cross‐gradient surficial aquifer site 720 and surface water sites 823 (pond), 749 (ditch downstream from a retention pond), and 747 (oxbow lake) were additionally sampled from.

The Riverton Wyoming Department of Energy (DOE) Legacy Management site is an example of an anthropogenic U contaminated freshwater aquifer, with former uranium and vanadium ore mill operations occurring from 1958 to 1963. During these operations, a 72‐acre tailings area with a 4‐ft depth of uranium, radium, and thorium containing waste was formed. 1.8 million cubic yards of this waste was relocated in 1988 to the Gas Hills East Disposal Site with surface remediation performed in 1989 (U.S. Department of Energy, Office of Legacy Management 2025). However, the surficial aquifer was contaminated with U levels exceeding background levels of 0.044 mg/L (Dam et al. 2015). Site levels are regularly monitored across the watershed and U is not flushing at the rate originally expected with detected concentrations varying before and after flooding events (Dam et al. 2015). A map of the site is depicted as Figure 1. Water flows in the surficial aquifer southeast towards the Little Wind River (U.S. Department of Energy, Office of Legacy Management 2025). The near‐river region is referred to as the “SSMA,” or St. Stephen's Mission Area. Once the water reaches the Little Wind River, U is diluted to below levels of human and ecological health concern and has low bioavailability (Byrne et al. 2021). An oxbow lake developed at the site in 1995 (U.S. Department of Energy 2024) which presents its own unique challenges for redox‐sensitive metal transport dynamics (Aeppli et al. 2022; Babey et al. 2022; Ghosh and Donselaar 2023). Since the closure of U milling on the site, a sulfuric acid plant has been in operation. The plant creates retention ponds of treated wastewater (U.S. Environmental Protection Agency 2020) slightly upgradient from the main U plume and near the former tailings area (FTA).

Uranium mobility at the Riverton site has been recently explored. The Riverton site has a natural flushing plan based on estimated flushing within 100 years per CFR Code 40 (CFR Code 40 1983), but recent models have suggested the flushing is not occurring at the rate expected (Dam et al. 2015). U remediation is often based on microbial reduction reactions to immobilize U (Bargar et al. 2013; Jeong et al. 2023; Wu et al. 2006); generally U(VI) is more soluble compared to reduced U(IV) (Smedley and Kinniburgh 2023). However, this strategy's long‐term efficacy is limited due to U(IV) reoxidation if reducing conditions are not maintained (Singh et al. 2013). Instead of the traditional reductive immobilization scheme, oxidative enhanced flushing was trialled at this site. The oxidative enhanced flushing entailed oxic bicarbonate‐poor injections followed by oxic bicarbonate‐rich injections (Applied Studies and Technology 2023). The theory was to mobilize reduced U through oxidation, which has been shown in a previous study as more successful under carbonate facilitation (Seder‐Colomina et al. 2018). Results from the trial showed that U, Fe, and Mn had differing aqueous concentrations than advection–diffusion‐dispersion tracer models alone could explain (Applied Studies and Technology 2023). These transition metals are microbially influenced through reduction and oxidation metabolisms, and microbial reactions can exceed the abiotic reaction rate (Chen et al. 2025) or bypass kinetic constraints (Nealson 2006). Furthermore, microbial reactions are shown to have secondary effects on U transport, for example, microbial dissimilatory nitrate reduction product nitrite oxidizing U(IV) (Westrop et al. 2023), or biogenic Fe(II)‐sulfides reducing U(VI) (Veeramani et al. 2013). However, it is unknown if microorganisms at this site are contributing to the unexpected U and transition metal responses to the oxic injections.

Riverton site microbial experiments have been limited to the SSMA region (Tolar et al. 2020) which we assert is not representative of the entire aquifer system. It is well‐established at other metal‐contaminated aquifer sites that microbial communities vary based on plume location, geochemistry, and compartment (sediment‐bound versus planktonic) (Flynn et al. 2013; Hug et al. 2015; Smith et al. 2015), so we hypothesized that Riverton microbial communities would have similar physical drivers of their composition. We also hypothesized that functional tests would display regional variations. Specifically, we predicted microbial community members with known reductive metabolisms would be located closer to the river where there is a naturally reduced zone, and more oxidizing metabolisms in the FTA region. To test the hypotheses posed, we performed microbial community sequencing and nine metabolism stimulation tests on 11 resident Riverton water microbial communities. We then combined these results with statistical analyses against geochemical data. Finally, we made predictive models of how these measured metabolisms interact with uranium. These experiments fill a site‐specific knowledge gap; the Riverton site has not been tested for cross‐site microbial membership nor metabolisms, and metal reduction–oxidation reactions, including microbial, are crucial to contaminant management strategy.

## 2 Materials and Methods

### 2.1 Materials and Reagents

Table S2 provides the full list of reagents used and their manufacturer and catalogue information.

### 2.2 Sampling

Three litres of unfiltered water were collected from multi‐channel wells 855 (at depths “2” and “3”), 859 (depth “3”), and 860 (depth “3”), surficial aquifer wells 710, 720, 784, and 789, and surface water sites 823 (pond), 749 (retention pond), and 747 (oxbow lake) (Figure 1) during regular groundwater sampling of the Riverton WY site in August 2023 as described in US DOE Verification Monitoring Report, Riverton, WY, Processing Site (U.S. Department of Energy 2024). Locations (elevation, latitude, longitude, and depth) are recorded in Table S1. Samples were collected into labelled sterile bottles alongside a shipping control with Lysogeny Broth medium and shipped overnight in coolers with ice to the University of Wisconsin‐Madison. Samples were stored at 4°C until use. No growth was noted in shipping control medium during sampling nor upon receipt of shipment.

### 2.3 Geochemical Measurements

Total groundwater iron concentration was determined using inductively coupled plasma optical emission spectroscopy (ICP‐OES, Agilent 5110 VDV) and an internal yttrium standard. Sample aliquots and calibrants were acidified with nitric acid (2%) prior to measurement. Fe concentration values were determined from Fe(II) chloride tetrahydrate calibrants (0.0900, 0.180, 0.890, 1.78, 3.11, and 4.44 ppm, *R* ^2^ value of 1.00) in 2% nitric acid. All sample, calibrant, and blank measurements were determined based on the 238.20 nm wavelength.

Metrics for total alkalinity (as calcium carbonate), pH, specific conductance, sulfate, temperature, turbidity, and metal concentrations (Mn, Mo, U) were acquired from the Department of Energy (DOE) Geospatial Environmental Mapping System (GEMS) database (Geospatial Environmental Mapping System (Gems), n.d.) (accessed June 12, 2024) as reported in the US DOE 2023 Verification Monitoring report (U.S. Department of Energy 2024).

### 2.4 Geochemical Modelling

PHREEQC version 3 (Parkhurst and Appelo 2013) was utilized to model U and Mn chemical distributions at sites with noted differences in metabolism results. Sites 789, 855–2, 860–3, and 747 had geochemistry compiled from 2015 to 2019 from GEMS (Geospatial Environmental Mapping System (Gems), n.d.), as those were the last dates with geochemical data surveyed for PHREEQC input requirements at these sites (Table S3). The database from Johnson et al. (2023) was referenced as it has updated Ca and Mn carbonate complexes.

### 2.5 Stimulation of Microbial Reduction–Oxidation Metabolisms Tests

#### 2.5.1 Metabolism Test Solutions and Stocks Preparation

To test the Riverton groundwater microbial community across the site for the capability of nine different energy metabolisms, raw groundwater was added to a defined medium and compared to autoclaved groundwater additions to the same medium. A base medium was designed by modifying the mineral, vitamin, and trace metal solutions from those described in Tanner (2007) to approximate conditions in the Riverton aquifer. Ion and metal concentrations were matched as feasible to averaged pre‐injection levels across wells 1020–1034 in September 2020 and wells 859 and 860 [multi‐depth] in June 2021 during field tracer testing at the site (Applied Studies and Technology 2023). These sites and dates were chosen as they contain the most recent detailed metrics for the surficial aquifer. The aquifer‐defined medium (ADM) had 0.239 g/L ammonium sulfate, 0.100 g/L monobasic potassium phosphate, 0.500 g/L magnesium sulfate heptahydrate, 0.120 g/L calcium chloride dihydrate, 0.083 g/L sodium metasilicate pentahydrate, and 0.203 g/L yeast extract in Milli‐Q (MQ) water. The trace metal solution contained 2.000 g/L nitrilotriacetic acid, 1.000 g/L manganous sulfate monohydrate, 0.700 g/L iron(II) chloride tetrahydrate, 0.200 g/L cobalt(II) chloride hexahydrate, 0.200 g/L zinc sulfate heptahydrate, 0.020 g/L copper(II) chloride dihydrate, 0.020 g/L nickel chloride hexahydrate, 0.060 g/L sodium molybdate dihydrate, 0.020 g/L sodium selenate, 0.020 g/L sodium tungsten oxide dihydrate, and 0.005 g/L sodium orthovanadate. Vitamin solution consisted of 10 mg/L pyridoxine‐HCl, 5 mg/L thiamine‐HCl, 5 mg/L riboflavin, 5 mg/L calcium pantothenate, 5 mg/L thioctic acid, 5 mg/L *p*‐aminobenzoic acid, 5 mg/L nicotinic acid, 5 mg/L vitamin B12, 5 mg/L mercaptoethanesulfonic acid, 2 mg/L biotin, and 2 mg/L folic acid in MQ water. Both supplement solutions were adjusted to pH 7 and filtered (Corning 431118, 0.22 μm PES).

Anoxic stocks for reductive reactions were prepared by purging with a 5% H_2_, 10% CO_2_, 85% N_2_ gas mixture, with 0.22 μm nylon filters (VWR 76479‐030) at the gas inlet and outlet of individual tubes. Solutions for oxidation tests were not purged.

Sodium bicarbonate, nitrate, Fe(III), and Mn(IV) stocks were autoclaved, while uranyl acetate was filtered (0.22 μm PES filter) and purged with a gas mix as described above to maintain anoxic conditions.

#### 2.5.2 Metabolism‐Specific Additions

Metabolism tests performed are summarized in Table 1, with test‐specific reactant and carbon additions alongside endpoint method described. Initial electron donor or terminal electron acceptor reactants were added in excess to stimulate metabolisms of interest and more favourable nutrient sources for assimilation were supplied in the media, as described in other literature (Tanner 2007; Gerhardt 1994; Cardenas et al. 2008). All media were autoclaved, and the post‐autoclave additions were 10.0 mL/L vitamin solution, 10.0 mL/L trace metal solution, 20.0 mL/L 1.00 M sodium bicarbonate (except DSR), and pH was adjusted to 7.1 ± 0.3. Reduction test media were purged with a 5% H_2_, 10% CO_2_, 85% N_2_ gas mixture.

**TABLE 1:** Metabolism‐specific stimulation additions and endpoint methodology.

Metabolism test	Abbr.	Reactant added	Carbon sources	Endpoint
Ammonium oxidation	AO	18.1 mM ammonium sulfate	0.080 g/L peptone, 0.24 mM sodium acetate	Quantitative: NH_4_^+^ depletion by IC
Mn(II) oxidation	MnO	0.12 mM manganous sulfate	0.080 g/L peptone, 0.24 mM sodium acetate	Quantitative: aqueous Mn concentration by ICP‐MS/MS
Sulfur‐species oxidation	SO	38 mM sodium thiosulfate	0.080 g/L peptone, 12 mM sodium acetate	Quantitative: SO_4_^2−^ formation by IC
Fe(II) oxidation	FeO	Bottom medium: 50 mM iron(II) chloride tetrahydrate	Bottom medium: 1.00 mM sodium acetate	Qualitative: Fe(III) banding pattern, gradient culture
Nitrate reduction	NR	10 mM sodium nitrate	20 mM sodium DL‐lactate, 10 mM sodium acetate	Quantitative: NO_3_^−^ depletion by IC
				Qualitative: Gas production
Mn(IV) reduction	MnR	20 mM manganese oxide	7.1 mM sodium DL‐lactate, 1.6 mM sodium acetate	Quantitative: Mn(IV) depletion by benzidine assay
Fe(III) reduction	FeR	8 mM iron(III) citrate	2.5 mM sodium DL‐lactate, 0.88 mM sodium acetate	Quantitative: Fe(II) production by ferrozine assay
U(VI) reduction	UR	2 mM uranyl acetate	2.5 mM sodium DL‐lactate, 0.88 mM sodium acetate	Quantitative: U(VI) depletion by spectrophotometry assay
Sulfate reduction	DSR	30.0 mM sodium sulfate	60.0 mM lactate, 1.00 g/L yeast extract	Quantitative: SO_4_^2−^ depletion by IC, Qualitative: Black Precipitates

Abbreviations: Abbr., abbreviation for metabolism test performed; IC, Ion chromatography; ICP‐MS/MS, inductively coupled plasma tandem mass spectrometry.

Ammonium oxidation (AO), manganese(II) oxidation (MnO), and sulfur‐species oxidation (SO) metabolism tests were prepared by the addition of reactant and carbon sources to 10.0 mL ADM in borosilicate culture tubes (18 × 150 mm), per Table 1. For SO metabolism test, concentration and usage of sodium thiosulfate to supply sulfide stably via thiosulfate disproportionation was based on Luo et al. (2013). Filter‐sterilized manganous sulfate was added post‐autoclave for the MnO test.

Fe(II) oxidation (FeO) was tested in gradient Fe(II)‐oxygen cultures based on procedures described in Sobolev and Roden (2001), Emerson and Merrill Floyd (2005), and Yli‐Hemminki et al. (2014) Briefly, a dual layer medium was prepared with the bottom layer containing ADM with 1.00 mM sodium acetate, 2% weight‐per‐volume agar, 20 mM sodium bicarbonate, and 50 mM FeCl_2_. The top layer was composed of ADM with 10.0 mL/L vitamin solution, 10.0 mL/L trace metal solution, and 20 mM sodium bicarbonate, creating a Fe(II) gradient of 0–50 mM. Agar tubes were prepared in an anaerobic chamber (Coy Laboratory Products, Grass Lake, Michigan, USA) with a 7% H_2_, 20% CO_2_, 73% N_2_ mixed gas. These solidified overnight in the anaerobic chamber, then were removed to aerobic laboratory settings to form the O_2_ gradient culture conditions. While pulling a pipette through the top layer, 20.00 μL of inoculum was injected. This was performed for each site in duplicate (replicates R1‐2), each site's autoclaved groundwater control, and a MQ water control.

Nitrate reduction (NR), Mn(IV) reduction (MnR), Fe(III) reduction (FeR), and U(VI) reduction (UR) metabolisms were tested in Hungate tubes (18 × 150 mm) using anaerobic ADM medium and condition‐specific sodium acetate, sodium DL‐lactate and reactants as prepared in 2.5.1, at ~pH 7.0 (Table 1). Nitrate usage was determined based on Cardenas et al. (2008), Westrop et al. (2023) and Fries et al. (1994), Fe(III) citrate usage by Cardenas et al. (2008) and Cardoso et al. (2023), and uranyl acetate usage by Jeon et al. (2004).

The sulfate reduction (DSR) metabolism test was performed in Hungate tubes (18 × 150 mm) using anaerobic MOYLS4 medium (Hou et al. 2024), prepared with 8.00 mM magnesium dichloride hexahydrate, 20.0 mM ammonium chloride, 0.600 mM calcium chloride dihydrate, 2.00 mM dipotassium phosphate, 2.00 mM monosodium phosphate, 1.00 g/L yeast extract, 15.0 mL/L 2.00 M Tris–HCl (pH 7.4), 0.060 mM FeCl_2_, 0.120 mM EDTA, 60.0 mM lactate, and 30.0 mM sodium sulfate anhydrous in MQ water.

#### 2.5.3 Inoculation of Metabolism Tests

All metabolism tests, except for FeO as described in 2.5.2, were inoculated with 1.0 mL of raw groundwater to replicate tubes upon first opening of sampling bottles in the laboratory. One milliliter of autoclaved groundwater matched to each site and 1 mL of autoclaved MQ water was added to control tubes. All inoculations were performed next to a flame. All metabolism tests were incubated at room temperature in the dark and were inverted or lightly vortexed three times per week.

#### 2.5.4 Endpoints of Metabolism Tests

Images were taken regularly throughout tests with 2–3 images/metabolism test/site/week. All qualitative endpoints were assessed at the 6‐weeks timepoint. Qualitative metabolism endpoints included formation of black Fe(II) sulfide precipitates for DSR metabolisms (Cardenas et al. 2008; Hou et al. 2024), gas formation for NR metabolisms (Cardenas et al. 2008), and formation of an Fe‐oxide ring in the gradient culture for FeO metabolisms (Sobolev and Roden 2001; Emerson and Merrill Floyd 2005; Yli‐Hemminki et al. 2014).

All quantitative endpoints were performed after 15 weeks to allow for slow growing microbial community members. NR, AO, SO, and DSR were determined quantitively by changes in anion or cation concentration, as listed in Table 1, by IC using a Dionex 4 mm ICS‐2100 and ICS‐1100 with mobile phases of 15 mM methanesulfonic acid and 3.5 mM sodium carbonate/1 mM sodium bicarbonate, a 25 μL loop size, AS14/CS12A columns, and ASRS 4 mm/CSRS_4mm suppressors. Sulfate concentrations were determined from peak area (μS × min) at ~9.4 min against a calibration curve (4.00 × 10^1^, 2.00 × 10^2^, 3.60 × 10^2^, 5.20 × 10^2^, 6.80 × 10^2^ ppm calibrants, *R* ^2^ value of 0.998). Ammonium concentrations were determined from peak area (μS × min) at 3.75 min against a calibration curve (6.00, 30.0, 54.0, 78.0, and 102 ppm calibrants, *R* ^2^ value of 0.999). Nitrate concentrations were determined from peak area (μS × min) at 6.43 min against a calibration curve (6.00, 30.0, 54.0, 78.0, and 102 ppm calibrants, *R* ^2^ value of 0.996). As needed, 10× or 20× dilutions for samples were performed.

Mn(IV) reduction was assessed quantitatively by the benzidine assay adapted from Burnes et al. (1998) Briefly, 50.0 μL of sample was added to 1.00 mL of 2.00 mM benzidine hydrochloride solution in 10% acetic acid. 200.0 μL of this was added to a plate reader, and the absorbance was read at 415 nm over 20 min at 30 s intervals. Maximum absorbance values were compared to calibrants prepared from Mn(IV) oxide (2.00, 5.00, 10.0, 20.0, 30.0 mM calibrants, *R* ^2^ value 0.996), and oxidation state selectiveness by benzidine was verified by a Mn(II) chloride tetrahydrate control.

The MnO metabolism was to be assessed quantitatively with the benzidine assay described for MnR metabolism. However, the benzidine assay was unable to accurately measure the lower Mn(IV) concentrations needed for this study; therefore, total aqueous Mn levels were instead determined by ICP‐MS/MS. Since measurement was taken after filtering, we assumed that the sparingly soluble Mn(IV) oxides were removed and only Mn(II) remained in the solution measured. ICP‐MS/MS (Agilent 1260 Infinity II) was performed with a 0.22 μm syringe‐filtered sample that was diluted (20×) and acidified (2% HNO_3_) against prepared manganous sulfate standards (10.0, 100, 200, 300, 400, 500. ppb, *R* ^2^ value 1.00). An ICP‐MS/MS internal yttrium standard was used. Each sample was measured in triplicate by the instrument with average and relative standard deviation (RSD) values as outputs.

FeR metabolism was assessed quantitatively by the ferrozine assay (Cardoso et al. 2023) for formation of Fe(II). Briefly, 100.0 μL of sample was added to 1.00 mL of 0.500 M HCl. 200.0 μL of this was added to 1.30 mL ferrozine solution (1.00 g/L ferrozine in 50.0 mM HEPES). After 10 min, the solution was filtered using a 0.2 μm syringe‐filter, and absorbance was read at 562 nm. Each sample was measured in duplicate, compared against Fe(II) standards (1.00 mM, 2.00 mM, 4.00 mM, 6.00 mM, 8.00 mM, 10.00 mM, *R* ^2^ 0.991) prepared with a FeCl_2_/EDTA stock, and values averaged.

UR metabolism was assessed by U(VI) spectrophotometric determination (Elnahas et al. 2021; Johnson and Florence 1971). Ten μL of sample was added to 100. μL complexing solution (25 g/L DCTA, 5 g/L sodium fluoride, 65 g/L sulfosalicylic acid, pH 7.85), 100. μL Tris buffer (pH 7.85), 500. μL ethanol, 100. μL PADAP (100 g 2‐(5‐Bromo‐2‐pyridylazo)‐5‐(diethylamino)phenol per 100. mL ethanol), and 350 μL MilliQ water. The mixture was incubated for 40 min, and absorbance was measured at 578 nm. Each sample was measured in duplicate, compared against U(VI) standards (0.20, 0.30, 0.50, 1.00, 2.00, and 3.00 mM, *R* ^2^ value 1.00) prepared from uranyl acetate, and the values were averaged.

Percent change for metabolism tests by disappearance of the reactant (AO, NR, MnO, MnR, DSR, and UR) was calculated with Equation (1) and then averaged for each site. The autoclaved control used in this calculation was site‐specific.

(1) $$100\times \left(\text{Autoclaved Control}-\text{Replicate}\right)/\text{Autoclaved Control}$$

Percent change for metabolism tests determined by appearance of product (FeR, SO) was calculated with Equation (2) and averaged for each site.

(2) $$100\times \left(\text{Replicate}-\text{Autoclaved Control}\right)/\text{Theoretical Change}$$

This was performed for FeR using a theoretical change of 8 mM, based on full conversion of added Fe(III). Theoretical change value for SO was 76 mM based on reactant addition of 38 mM sodium thiosulfate (Na_2_S_2_O_3_) converting to SO_4_^2−^.

Percent change for binary FeO test was performed by averaging the binary result (0 or 1) and multiplying that by 100. This resulted in values of 0, 50, or 100 for FeO percent change options, where 0 corresponds to no Fe oxidation detected, 50 to one replicate with FeO detected, and 100 to both replicates with FeO detected.

### 2.6 DNA Extraction and Amplicon Community Sequencing

Approximately 1 L of water from each site was filtered through a 0.1 μm PES membrane filter (ThermoScientific Nalgene RapidFlow Lot 1,376,832). The filter membrane was removed under sterile conditions and frozen at −80°C. Filters for sites 747 and 823 were split in further extraction and sequencing steps. DNA was extracted using Qiagen DNeasy PowerWater Kit instructions. DNA concentrations were read via a Qubit 3 Fluorometer (Invitrogen by ThermoFisher Scientific) using broad range solutions (1× dsDNA BR assay kit, ThermoFisher). DNA yield was insufficient for metagenomics and metatranscriptomics analyses, so 16S amplicon sequencing was instead performed.

Amplicon library preparation with PCR‐amplification targeting the V4 region of the 16S rRNA gene and sequencing on an Illumina Miseq was performed according to our established methods (Chatman et al. 2024). Briefly, primers used were dual‐indexed designed using high‐fidelity polymerase (Kozich et al. 2013), Pfx. A negative control and positive control (Pfx) were added during each sequencing run. Gel electrophoresis was performed verifying amplified PCR products. Amplified products were normalized to 20 μL using a SequalPrep Normalization kit (Life Technologies) and aliquots of 5 μL from normalized samples were pooled into the final library. Final concentrations were verified using KAPA library quantification kit (Kapa Biosystems) and a Qubit 2.0 Fluorometer (Invitrogen). Then, the final library was diluted to 20 nM with HT1 buffer and PhiX control v3 (20% vol/vol), and 600 μL was loaded onto a MiSeq v2 (500 cycles) reagent cartridge (Illumina). Sequences were uploaded to Illumina Sequence Hub.

### 2.7 Microbial Community Analyses

Sequencing files were accessed using BaseSpace Sequence Hub Downloader (Illumina) and processed for bioinformatics via Quantitative Insights into Microbial Ecology (QIIME) 2 (version 2024.5) (Bolyen et al. 2019). Data was uploaded to Qiime2 using Casava 1.8 paired‐end demultiplexed format (via Qiime import tools). DADA2 (Callahan et al. 2016) denoise‐paired action was used to trim and truncate sequences (values chosen based on demux file interactive quality plot for below 8 or past the 30% quality control threshold), denoise, and quality filter (with chimera consensus method). Amplicon sequence variants (ASVs) were aligned using MAFFT (Katoh and Standley 2013) and FastTree (Price et al. 2010) (both via Qiime‐phylogeny). Taxonomic assignments were made with a Naïve Bayes Classifier, trained by Sklearn 1.4.2 on 05/30/2024 using Silva 138 99% operational taxonomic unit full‐length sequences (provided from Qiime2) (Robeson II et al. 2021; Bokulich et al. 2018; Quast et al. 2013).

Qiime2‐processed files were uploaded in R (version 4.4.0) (R Core Team 2024) using qza_to_phyloseq via qiime2R. These were then processed and compositional barplots visualized using package microViz (Barnett et al. 2021). Alpha diversity was calculated using phyloseq (McMurdie and Holmes 2013) function estimate_richness for Shannon's diversity index. Beta diversity was performed with vegan (Oksanen et al. 2025) function metaMDS for Bray–Curtis dissimilarity index for two dimensions (*k* = 2) and visualized with ggplot as part of tidyverse (Wickham et al. 2019) with metadata of water type and location descriptors added.

### 2.8 Correlation Analyses

Spearman correlations were calculated and visualized in R using the microViz package (Barnett et al. 2021) for ASVs (Table S5) against geochemical values (Table 2) and percent change values (Table S7). ASVs were prefiltered such that replicate samples were removed and ASVs were required to be present in three sites for correlation analysis.

**TABLE 2:** Geochemical values for sampling sites.

Site code	Location descriptor	Total alkalinity as CaCO_3_, ppm	Mn, ppm	Mo, ppm	pH	Specific Conductance, μmhos/cm	Sulfate, ppm	Temp °C	Turbidity, NTU	U, ppm	Fe, ppm^a^
Groundwaters											
710	Upgradient	188	0.0184	0.00311	7.27	719	146	11.57	0.29	0.00523	0
720	Crossgradient	226	0.002	0.00201	7.21	568	78.2	13.5	0.53	0.00386	0
784	FTA	370	0.79	0.167	7.26	4161	1830	16.96	1.62	0.0137	0.02^b^
859–3	FTA	305	1.03	0.0965	6.84	6047	3280	15.3	3.47	0.145	3.19
860–3	FTA	248	1.44	0.26	6.82	3741	1930	15.34	1.11	0.981	1.64
855–2	SSMA	435	0.441	0.309	7.08	9240	5030	15.11	1.86	0.825	2.28
855–3	SSMA	388	0.839	0.329	7.03	8336	4420	15.26	2.92	0.755	0.09
789	SSMA	365	0.0822	0.491	7.01	7927	4375	12.73	0.65	0.8655	0.02^b^
Surface waters											
823	Upgradient	95	0.0533	0.000673	8.78	2757	966	27.28	3.82	0.00298	0.01^b^
749	Retention Pond Ditch	179	0.0172	0.00455	7.17	954	225	20.59	6.4	0.000137	0.91
747	Oxbow Lake	323	0.698	0.0185	9.33	1349	321	18.38	13.1	0.101	0.09

*Note:* Iron values were determined from ICP‐OES, and all other metrics were compiled from the 2023 Verification Monitoring Report (U.S. Department of Energy 2024) and the Geospatial Environmental Mapping System (GEMS) Legacy Management database (Geospatial Environmental Mapping System (Gems), n.d.).

^a^ Parameter in Table 2 newly measured in this study. Other parameters are reported as stated in GEMS (Geospatial Environmental Mapping System (Gems), n.d.).

^b^ Value was below the lowest calibrant (at 0.09 ppm), but the area under the curve at wavelength 238.20 nm was detectable and above the blank value.

## 3 Results

### 3.1 Riverton Geochemistry

Select geochemical measurements made here, and in database reports (Geospatial Environmental Mapping System (Gems), n.d.), are reported in Table 2 (Geospatial Environmental Mapping System (Gems), n.d.). U was elevated above the site maximum contaminant limit of 0.044 mg/L at FTA sites 859–3 and 860–3, all groundwater SSMA sites, and oxbow lake site 747. Aqueous Mn and Mo were detectable in all sites; however Mn and U were highest at FTA site 860–3 whereas Mo was highest in SSMA sites. Fe concentrations in the groundwater samples varied and were undetectable in 710 and 720, whereas within the plume, concentrations ranged from 0.02 to 3.19 mg/L. Sulfate levels ranged across the site with upgradient and cross‐gradient sites at 146 and 78.2 mg/L, respectively, and plume groundwater concentrations elevated, ranging from 1830 to 5030 mg/L. pH was roughly circumneutral within groundwater samples, ranging from 6.82 to 7.27, and slightly elevated in upgradient surface water and the oxbow lake. Turbidity was highest at oxbow lake site 747 and lowest at upgradient pond site 710. These results show the Riverton site as pH circumneutral with high sulfate concentrations and varying metal concentrations based on the U plume.

PHREEQC (Parkhurst and Appelo 2013) was utilized to determine U speciation for SSMA sites 789 and 855–2, FTA sites 859–3 and 860–3, and oxbow lake site 747 based on site archived geochemistry data (Table S3). Sites were chosen for analysis based on metabolism test results discussed later. Distributions of U species showed oxidation state 6 calcium uranyl carbonates primarily at all sites, and Mn as oxidation state 2 (Table S4).

### 3.2 Riverton Microbial Community

**FIGURE 2:**
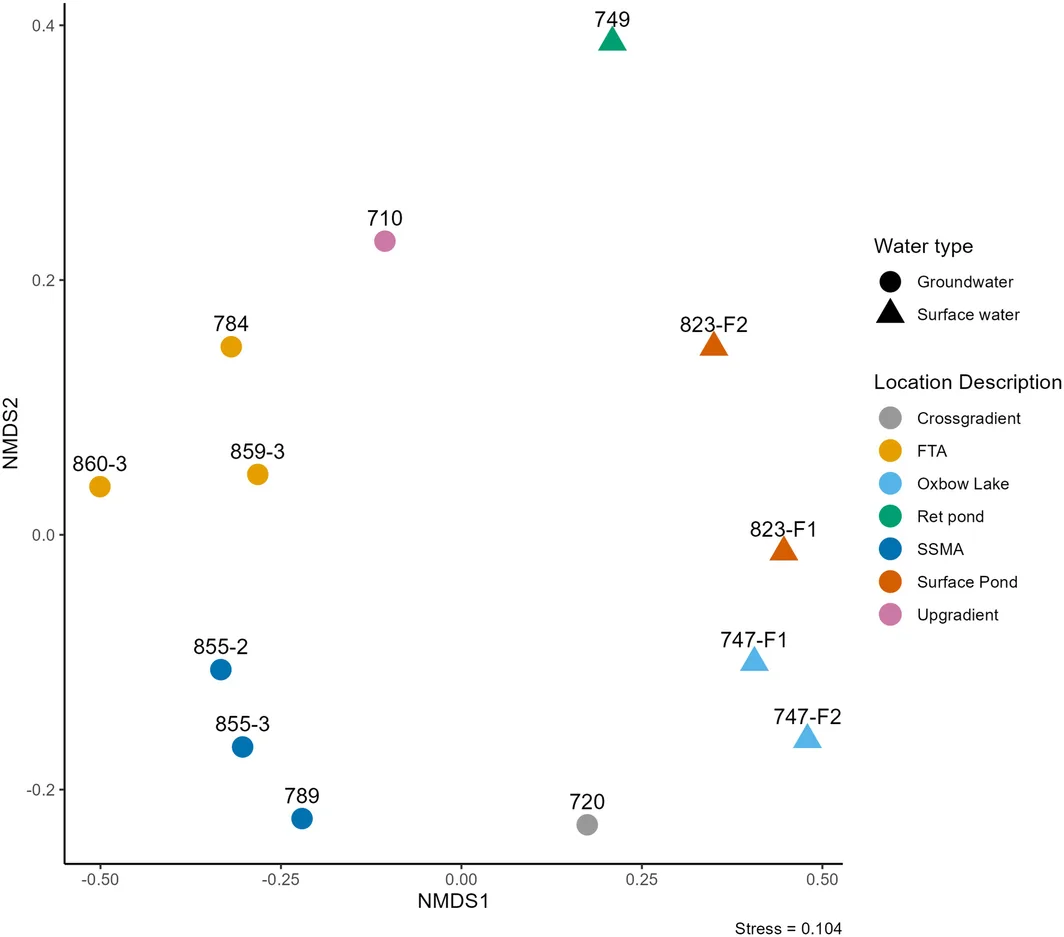
Non‐metric multidimensional scaling (NMDS) plot of Bray–Curtis dissimilarity β diversity results comparing the water microbial communities across the Riverton site. Descriptors used for site locations are indicated by colour, and groundwater versus surface water by shape. Descriptors include former tailings area (FTA), St. Stephen's Mission Area (SSMA), and surface water ditch nearest the retention pond (Ret pond).

A total of 1076 unique ASVs were identified in the water from the 11 Riverton well sites sampled (Table S5). Observed amplicon sequence variant (ASV) values per site varied from 69 to 151 (Table S6) and total counts per location ranged from 2050 to 7378 (Table S5). FTA sites had higher alpha diversity than SSMA sites based on Shannon and Simpson metrics, despite the observed number of ASVs for SSMA sites being within the range of FTA site values (Table S5). Metric Bray–Curtis dissimilarity was used to compare microbial communities across the Riverton samples (Figure 2). Communities ordinated across the x‐axis based on surface versus groundwater locations. Samples within subgroups FTA and SSMA were more similar to each other than to other locations, although most samples were not strongly clustered together. Replicates from surface water sites 823 and 747 were relatively similar to each other rather than to the groundwater sites. Notably, the retention pond ditch site 749 was furthest from other samples, suggesting higher dissimilarity in the planktonic community of the retention pond ditch from nearby groundwater region FTA.

**FIGURE 3:**
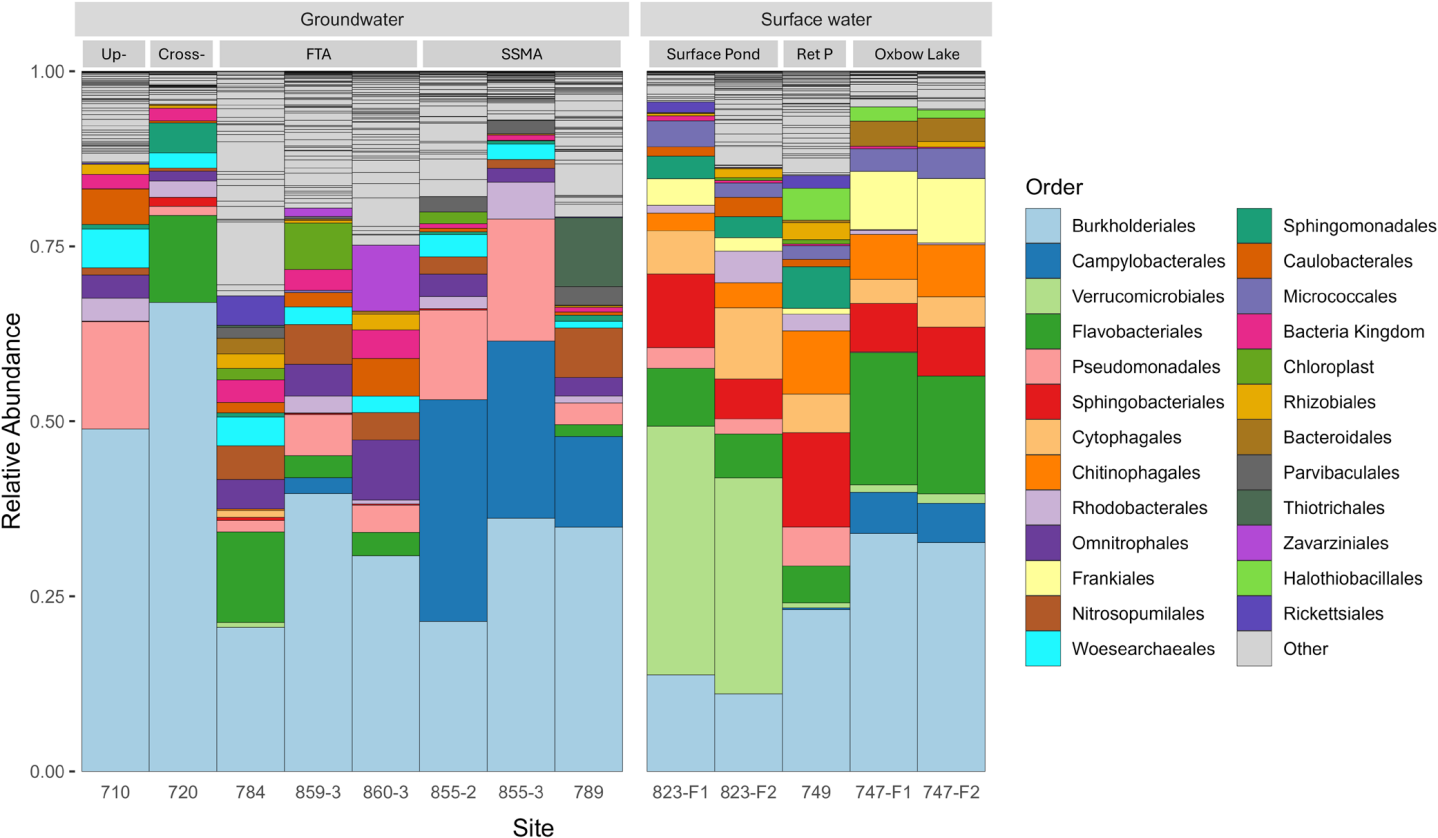
Relative abundance of the microbial community members from across the Riverton site. The top 25 orders identified by 16S amplicon sequencing are displayed. Samples are sorted by groundwater (surficial aquifer) and surface water samples. Groundwater samples are further sorted by their relationship to the U plume, with upgradient, cross‐gradient, former tailings area (FTA), and St. Stephen's Mission Area (SSMA) zones labelled. Surface water sites include two replicates of the upgradient surface pond and downgradient oxbow lake site and one replicate of the retention pond ditch near the FTA zone.

Microbial community membership was compared between samples (Figure 3, Table S5) and some taxonomic orders were found throughout all sampling locations. At the order level, ASVs identified as Burkholderiales were present in all groundwater and surface water sites. This was mainly the family Comamonadaceae. Order Pseudomonadales was identified in all sites, although minimally present in site 747. Families of this order included Cellvibrionaceae, Moraxellaceae, and Pseudomonadaceae among others, but no family within the order was identified throughout. Order Flavobacteriales was found in all regions (FTA, SSMA, upgradient, crossgradient, surface waters) but was missing from SSMA sites 855–2 and 855–3. Order Rhodobacterales was found in all regions as well but was missing from FTA site 784. Sequencing results showed similarities across locations for some taxonomic orders, but few lower classifications within these orders were the same between sites.

Surface waters had differing microbial communities compared to groundwaters (GW). Surface waters (SW) had higher relative abundances of Sphingobacteriales (SW 7%–13%, GW 0%–1.3%), Cytophagales (SW 3%–10%, GW 0%–1%), Chitinophagales (SW 3%–9%, GW 0%–0.2%), Frankiales (SW 1%–9%, GW 0%), and Micrococcales (SW 2%–4%, GW 0%–0.3%). The Sphingomonadales order was present in the upgradient pond (3%) and ditch near the retention pond (6%), although this order was not present in the oxbow lake and minimally in GW (< 1%). Groundwaters presented higher relative abundances than SW of orders Woesearchaeales (1%–6% GW, 0% SW), Omnitrophales (1%–9% GW, 0%–0.1% SW), and Nitrosopumilales (0.5%–7% GW, 0% SW).

Regionally the microbial community varied. In the SSMA, ASVs identified as order Campylobacterales are present in both groundwater (sites 855–2, 855–3, and 789) and the surface water of the oxbow lake (747‐F1 and 747‐F2), whereas this order was minimally present in the FTA. The relative abundance between groundwater and surface water of Campylobacterales varied though, with groundwater SSMA sites at 13%–32% relative abundance compared to the oxbow lake at 6%. FTA and upgradient groundwater site 710 had higher values of Caulobacterales than SSMA sites (1.5%–5% compared to SSMA at 0.1%–0.5%) and Rhizobiales (0.4%–2%, SSMA 0%–0.2%). Desulfitobacteriales was present in FTA sites 784 and 859–3 and oxbow lake site 749 but was not present in the other samples.

Sites also had specific orders associated with them. For example, Thiotrichales (identified at genera level as *Thiothrix*) was present at 10% in site 789 but less than 0.3% in all other sites. Halothiobacillales (identified at genera level as *Thiovirga*) is present at 5% in site 749, minimally present (1%–2%) in site 747, and not present in any other sites. Rickettsiales was present at 4% in site 784, minimally present in sites 823 and 749 (1.5% and 1.8%, respectively), present at < 1% in sites 710 and 789, and 0% at other sites. Lachnospirales was present at 9% in site 784, present at < 1% in sites 859–3 and 789, and not present in any other sites. Thalassabaculales (identified at genera level as *Thalassobaculum*) was present at 4% in site 860–3, 0.3% in 855–2, and undetected in other sites. Many other orders were detected with minimal presence across the site or were dispersed at various locations, for example, CHAB‐XI‐27, Legionellales, Reyranellales, Pirellulales, Desulfobulbales, and so forth. 16S amplicon sequencing of the microbial community shows variability based on water type and geographical location, with sites closer together having some underlying similarities but each with distinct microbial membership.

### 3.3 Correlation of Geochemistry and Microbial Community Composition

**FIGURE 4:**
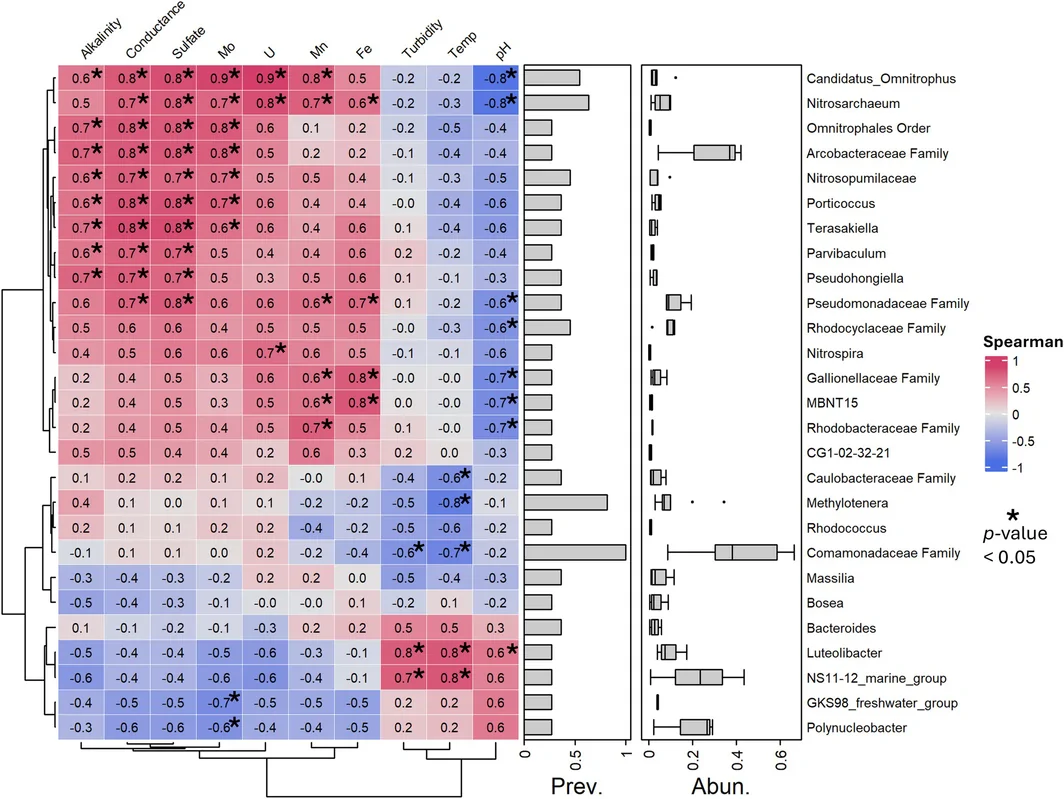
Spearman correlations of the most prevalent amplicon sequence variants (ASVs) and geochemistry. The value of each correlation is shown and coloured, with positive correlations as red and negative correlations as blue. Prevalence and abundance of each ASV is shown, and ASV are described on the genera level or the lowest identifiable level. *p*‐values from the Spearman correlation with a value less than 0.05 are marked with an asterisk.

To further analyse how the planktonic microbial composition may vary based on site geochemistry, a Spearman correlation was performed. Multiple microbial members were found to have significant positive correlations with alkalinity, conductance, sulfate, Mo, U, Mn, and Fe (Figure 4). These same microbial members were negatively correlated with pH, with genera *Candidatus Omnitrophus, Nitrosarchaeum, Nitrospira*, and *MBNT15* showing significance. ASVs without genera resolution identified as families Psuedomonadaceae, Rhodocyclaceae, Gallionellaceae, and Rhodobacteraceae similarly demonstrated significant positive correlations with metal abundance and negative correlations to pH.

### 3.4 Microbial Metabolism Tests

**FIGURE 5:**
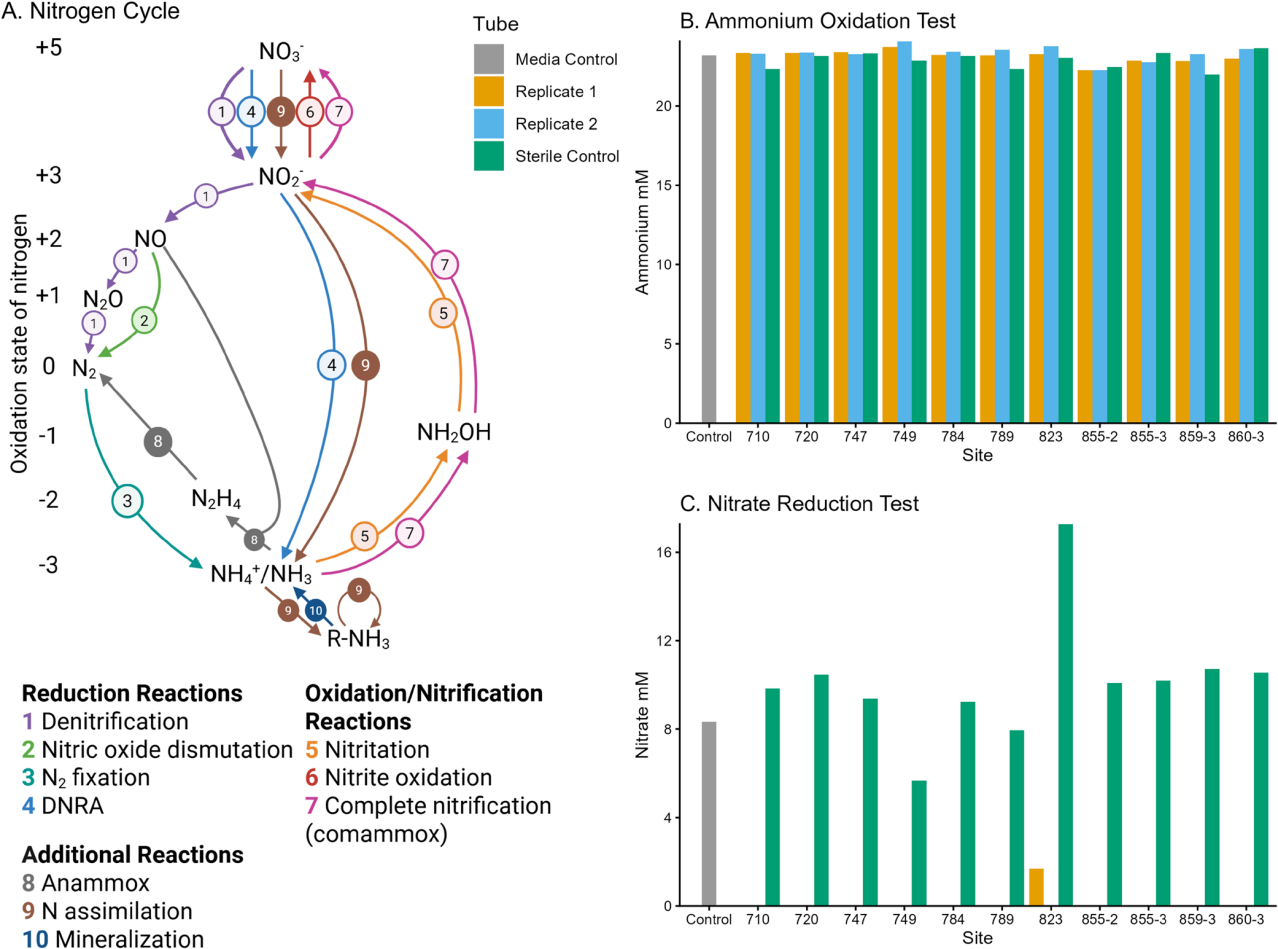
Nitrogen cycle metabolism results. (A) Nitrogen cycle as adapted from Zhang et al. (2020a, 2020b) and Kuypers et al. (2018), displaying microbial transformation reactions of major environmentally‐relevant nitrogen oxidation states (+5, +3, +2, +1, 0, −1, −2, −3) and generalized assimilated N (R‐NH_3_) which can exist as various oxidation states. (B) AO metabolism test results with ammonium concentration in mM. (C) NR metabolism test results with nitrate concentrations in mM. Bars not shown are levels of nitrate undetectable by ion chromatography.

Metabolism tests were performed to determine the potential for Riverton microbial communities to carry out metabolisms and uncover any variability in functional potential. Ammonia/um oxidation (AO) occurs through sequential oxidation steps of ammonia or ammonium, depending on the pH, to nitrite then to nitrate (Figure 5A, reactions 5–7). The measured ammonium concentration differences between controls and site‐specific replicates were small, with all values ranging from 22 to 24 mM ammonium (Figures 5B and S1A) and percent change values within ±5% (Table S7). This suggests that ammonium oxidation did not occur in these tests, that assimilation of ammonium was small to negligible (Figure 5A, reaction 9), or that rates of ammonium oxidation and assimilation were matched by those of ammonium‐producing reactions from other members of the microbial community, for example, N_2_ fixation, DNRA, or mineralization (Figure 5A, reactions 1–4). AO tests were performed aerobically, so it is unlikely that DNRA occurred in the presence of oxygen as a terminal electron acceptor or that anammox occurred. N_2_ fixation is suppressed by ammonium and oxygen, so that process was also likely to be negligible.

Nitrate reduction (NR) includes denitrification (Figure 5A reaction 1) and dissimilatory nitrate reduction to ammonium (DNRA, Figure 5A reaction 4). It is also feasible that nitrate can be assimilated (Figure 5A, reaction 9); however, ammonium in the medium was provided as a more direct N source for assimilation (Stevens et al. 2024). NR was tested quantitatively by nitrate disappearance from the medium (Figure S1B) and qualitatively by gas production (Cardenas et al. 2008). Results (Figure 5C) show complete nitrate depletion at every site except site 823, which had a 95% change of nitrate (Table S7). Gas production occurred in all R1 and R2 tubes. Notably, nitrate concentrations varied across site autoclaved controls, with surface water site 823 being almost double the concentration of other locations. The results demonstrate cross‐site microbial potential of nitrate reduction across the Riverton site samples.

**FIGURE 6:**
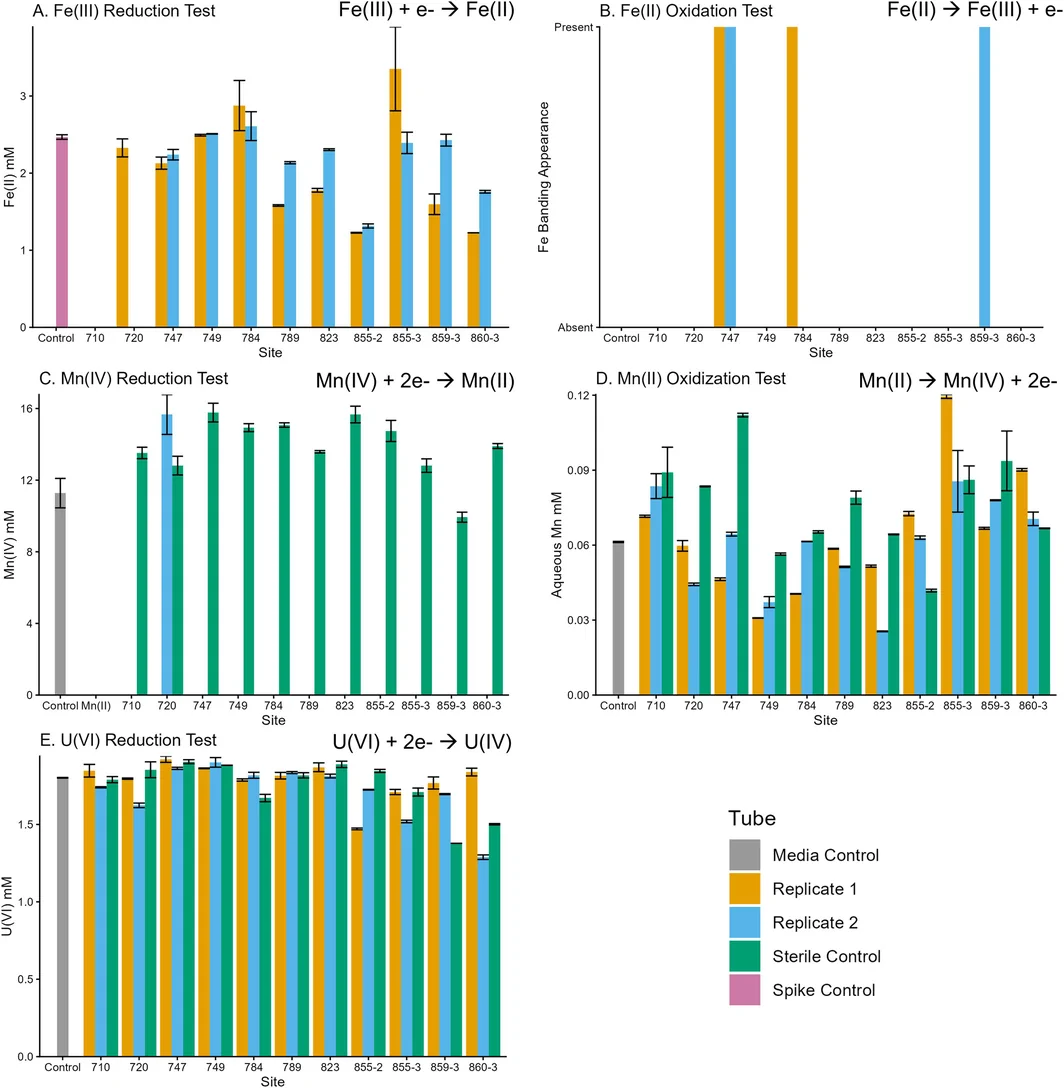
Metal metabolism results for Fe, Mn, and U. (A) Fe reduction metabolisms were measured by Fe(II) concentration. (B) Fe oxidation metabolisms were binary presence/absence data from banding appearance in Fe(II)–O_2_ gradient cultures. (C) Mn(IV) reductions were based on Mn(IV) concentrations. (D) Mn(II) oxidations were assessed based on aqueous Mn concentration. Error bars for aqueous Mn represent relative standard deviations from inductively coupled plasma tandem mass spectrometry results. (E) U(VI) reduction tests were based on U(VI) concentrations. Error bars present in Fe(III), Mn(IV), and U(VI) plots represent technical replicates of spectrophotometric assay results. Bars not plotted were values less than assay calibrant range.

Fe(III) reduction (FeR) metabolism was tested via measurement of Fe(II) production (Figure S1F). Fe(II) was produced in most sites except for one replicate from cross‐gradient groundwater site 720, upgradient groundwater site 710, and the autoclaved controls (Figure 6A). The ADM contains trace amounts of Iron(II) chloride to provide for assimilation needs, and since Fe(II) was not detected in the controls, it suggests that this supplied Fe(II) contribution to the FeR measurement was negligible. This provides evidence that sites within the groundwater U plume are capable of FeR.

Fe(II) oxidation (FeO) metabolism was tested through a gradient culture (Figure S1E). Results were qualitative based on formation of a reddish‐brown Fe band compared to controls with a gradient as described in previous literature (Sobolev and Roden 2001; Emerson and Merrill Floyd 2005; Yli‐Hemminki et al. 2014). Fe bands were observed in both replicates of oxbow lake site 747 and one replicate of FTA sites 784 and 859–3 (Figures 6B and S1E). This suggests that the Riverton microbial community across the site had a limited capacity for FeO in the conditions tested.

Mn(IV) reduction (MnR) metabolism was measured by Mn(IV) disappearance (Figure S1D). MnR results (Figure 6C) show complete Mn(IV) disappearance in all sites and replicates. The one exception was a replicate in the cross‐gradient of U plume groundwater site 720. The autoclaved controls showed little to no disappearance of Mn(IV), as expected (Figure 6C, green bars). Mn for assimilation was provided in the ADM as manganese(II) sulfate, so Mn(IV) disappearance can be attributed to energy metabolism.

Mn(II) oxidation (MnO) metabolism was tested through disappearance of Mn(II) (Figure S1C). MnO results (Figure 6D) had variability between replicates, with some replicates exceeding aqueous Mn concentrations of the autoclaved controls. The amount of Mn measured in the autoclaved controls across all samples also varied more than would be expected given the concentration in Riverton groundwater used from each site. This indicated that MnO may have occurred but is potentially obscured by active cycling between Mn(II) and Mn(IV) or assimilation of Mn. Therefore, MnO metabolism specifically was unable to be assessed in the methodology utilized herein.

U(VI) reduction (UR) metabolism was tested through the disappearance of U(VI) (Figure S1G). Some replicates show a small decrease relative to autoclaved controls (Figure 6E), but only site 855–2 had both replicates positive for UR, whereas in 859–3 both replicates had higher concentrations of U(VI) than the control at the end point, suggesting possible oxidation. Therefore, microbial UR metabolism was not overtly demonstrated by the planktonic microbial community across the Riverton site.

**FIGURE 7:**
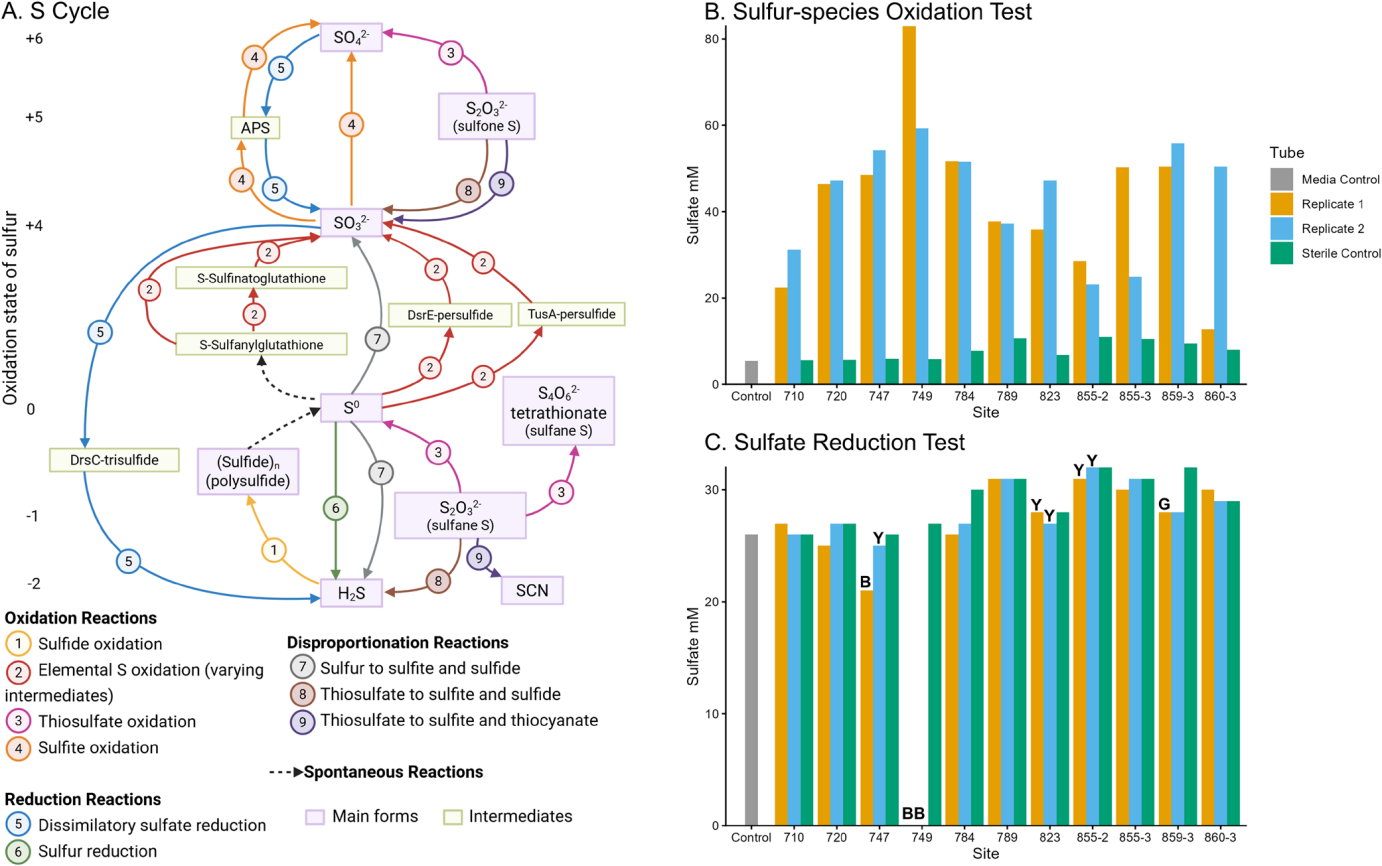
Sulfur cycle metabolism results. (A) The S cycle, simplified from Zhou et al. (2025), displays microbial oxidation, reduction, and disproportionation reactions of major environmentally‐relevant sulfur oxidation states (+6, +5, +4, 0, −1, −2). (B) Sulfur‐species oxidation (SO) metabolism test results are shown with sulfate concentration in mM. (C) Sulfate reduction (DSR) metabolism test results are shown with sulfate concentration in mM. Bars not shown are levels of sulfate undetectable by ion chromatography. Qualitative results are indicated for DSR metabolism with “B” indicating black precipitates, “Y” yellow precipitates, and “G” grey colouring of media.

Sulfur‐species oxidation (SO) was tested quantitatively by production of sulfate compared to autoclaved controls (Figure S1H). Thiosulfate was added in excess, which can be oxidized to sulfate, elemental sulfur, or tetrathionate (Figure 7A, reaction 3) or undergo disproportionation to sulfite and sulfide (Figure 7A, reaction 8). From these various sulfur species, sulfur can be microbially oxidized in multiple ways to the fully oxidized form of sulfate as described in Figure 7A. In all sulfur‐species oxidation metabolism replicates, sulfate production occurred and sulfate levels exceeded those of controls (Figure 7B). These results show microbial capacity for sulfur‐species oxidation across the site. The amount of sulfate detected may underestimate sulfur‐species oxidation that occurred since some SO pathways end at intermediates before the fully oxidized form, sulfate.

Sulfate reduction (DSR) was tested quantitatively by a decrease in sulfate concentration (Figure S1I). As noted in Figure 7A, sulfate is reduced by DSR metabolism to sulfite then to hydrogen sulfide. There were notable drops in sulfate concentration in samples 747, 749, 784, and 859–3 (Figure 7C). These had average sulfate percent‐change values of 12%, 100%, 12%, and 12%, respectively (Table S7). Most other sites had small DSR values (~2%), possibly suggesting the amount of sulfate disappearance that could be attributed to assimilatory sulfate reduction. DSR was also assessed qualitatively by the appearance of black Iron(II) sulfide precipitates. There was distinct blackening of the medium by the 6‐week timepoint of 747 Replicate 1 and 749 both replicates, and there was slight greying of 859–3 replicate 1 (Figure S1I). Since only Fe(II) was supplied in the ADM, this negates FeR in these tubes. Yellow precipitates formed in 747 replicate 2, 823 replicate 2, and 855–2 both replicates (Figure S1I). Sites 710, 720, 784, and 860–3 did not have DSR metabolism noted through qualitative nor quantitative metrics.

Metabolism results showed the microbial community across the site to be capable of nitrate reduction (NR), Mn reduction (MnR), and sulfur‐species oxidation (SO). Most communities were also capable of Fe reduction (FeR). No sites were shown as capable of direct, complete U(VI) reduction (UR) nor ammonium oxidation (AO). Differing sites were capable of sulfate reduction (DSR) and Fe oxidation (FeO).

### 3.5 Correlational Test of Microbial Community Membership and Metabolism Endpoints

**FIGURE 8:**
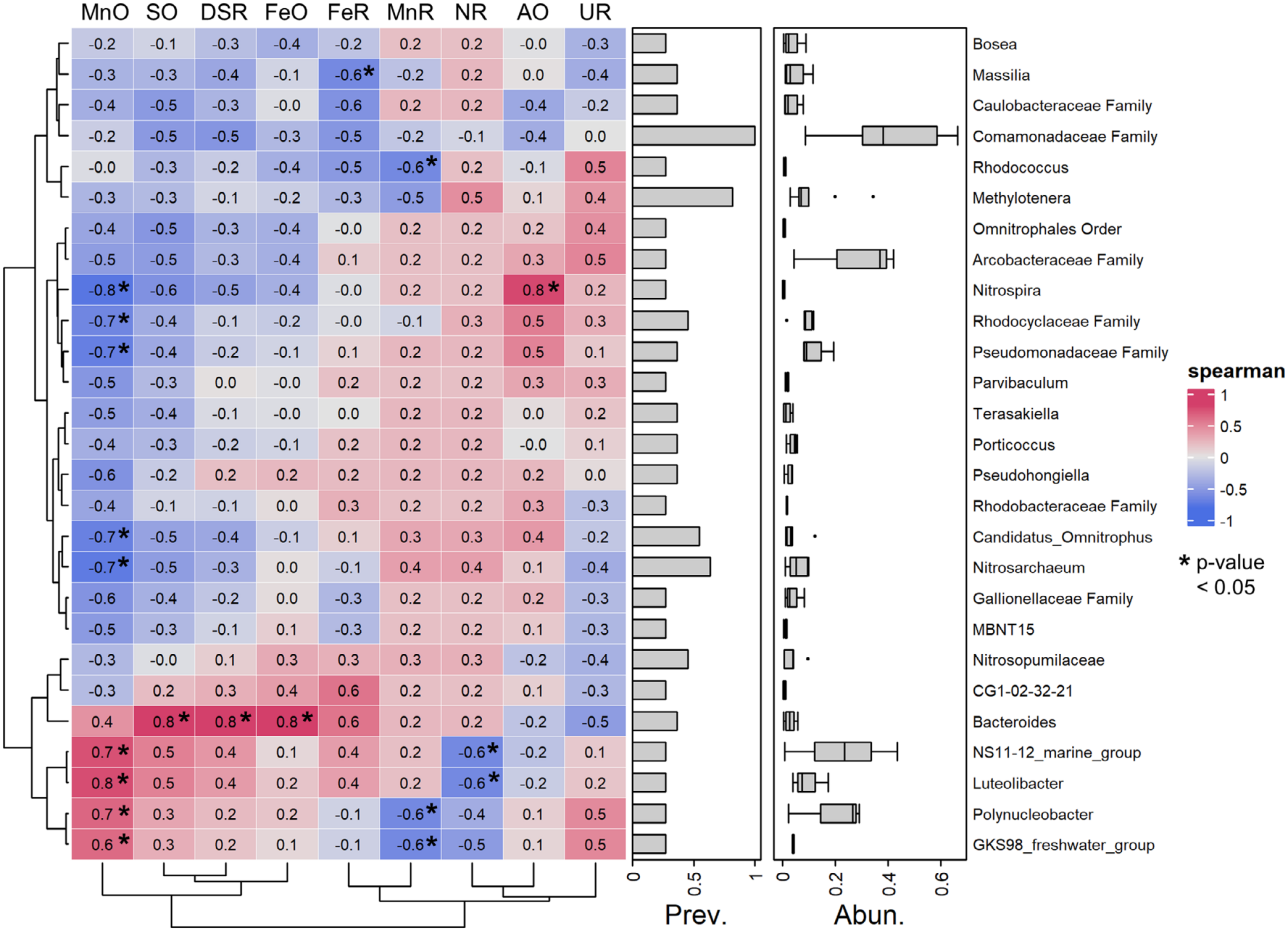
Spearman correlation between reduction–oxidation metabolism test percent change values and amplicon sequence variant (ASV) abundance. Samples 747 and 823 s replicates were removed and ASVs were filtered to be present in at least three samples for consideration. The value of each correlation is shown and coloured (red‐blue) based on value. Prevalence (Prev.) and abundance (Abun.) of each ASV is shown, and ASV are described on the genera level or the lowest identifiable level. *p*‐values from the Spearman correlation with a value < 0.05 are marked with an asterisk.

To test if measured amounts of different microbial metabolisms were related to the abundances of microbial community members, a Spearman correlation was performed (Figure 8). *Bacteroides*, an obligate anaerobe, had significant positive correlations with SO, DSR, and FeO metabolisms. Although MnO had the highest number of significant correlations between metabolism results and ASVs, the percent change (Table S7) values of MnO metabolism were inconsistent between replicates and against controls.

There were also some significant negative correlations noted, which indicated that the presence of these microbes may be inhibitory to the respective metabolism, for example, *Massilia*, *Polynucleobacter* and *GKS98 freshwater group* to Fe reduction, *Rhodococcus* to Mn reduction, and *NS11‐12 marine group* and *Luteolibacter* to nitrate reduction. Otherwise, there were slight but insignificant correlations (*p* > 0.05) with other ASVs and metabolisms. The strength of most correlations in Figure 8 is lower than geochemical‐ASV correlations in Figure 4.

## 4 Discussion

### 4.1 Microbial Community Membership Varies Across the Riverton Site Based on Location, Compartment, and Geochemistry

The Riverton, WY former uranium processing site has been geochemically (Sultana et al. 2024; Dangelmayr et al. 2023) and hydrologically (U.S. Department of Energy 2024; Paradis et al. 2022) characterized, results of which have driven site modelling (Perzan et al. 2021) and remediation plans (Applied Studies and Technology 2023). There were a few studies that investigated specific microbial reactions of interest (Cardarelli et al. 2020) or assessed near‐river region SSMA microbial community by depth (Tolar et al. 2020), however, microbial community membership and reactions have not been assessed throughout the site.

In measuring and comparing microbial membership across the former tailings area (FTA), near‐river region (SSMA), upgradient, cross‐gradient, and surface waters, the results validated the hypothesis of microbial community variation across the Riverton site. There were only 27 of the 1076 ASVs found that fulfilled the requirement of presence in 3 sites (Figure 4). The dispersed beta diversity (Figure 2) further indicated heterogeneity of the microbial community. This is also supported by the striking differences at various taxonomic levels between surface waters and groundwater, between regions of the aquifer, and between sites, as visualized in the taxa bar plots (Figure 3) and discussed in Section 3.2.

The planktonic community was identified as having different relative abundances from the soil/sediment‐bound community as observed by Tolar et al. (2020). Specifically, the Riverton SSMA region soil microbial community depth profile from the topsoil through the aquifer identified Proteobacteria as comprising 43%–50% of the total community, within which Deltaproteobacteria was the predominant class. Since that article has been published, the Deltaproteobacteria class has been split from the Proteobacteria phylum and four new phyla are now considered: Desulfobacterota, Myxococcota, Bdellovibrionota, and Desulfobacterota (Waite et al. 2020). Because of this rearrangement, the comparison of Proteobacteria levels and abundances found in this study is not as direct. The relative percentage of planktonic Proteobacteria measured in an SSMA region near the location of the previous study soil profile had similar abundance, ranging from 48% to 64% (Figure 3), despite Deltaproteobacteria now being considered a separate phylum. Phylum level differences between the aqueous planktonic (this study) and soil (Tolar et al. 2020) communities are also notable for Bacteroidetes, which ranged from 4% to 8% in SSMA region soil (Tolar et al. 2020) whereas in the SSMA groundwater values were much smaller, between 0.2% and 1.8% (Figure 3, Table S5). Although the number of studies and samples is limited, this suggests that in addition to differences in the microbial community being driven by geochemistry and region, the microbial community within one location has different membership in the planktonic aqueous phase and the attached phase within soils or sediments, consistent with other study results (Hug et al. 2015).

There are multiple significant positive correlations between ASVs and geochemical variables alkalinity, conductivity, sulfate, Mo, U, Mn, and Fe (Figure 4). Many of these same ASVs had negative correlations with pH. Another Riverton‐site specific publication also reported community variability based on covarying factors of depth, moisture, and metals concentrations at near‐river sampling cores (Tolar et al. 2020). Other uranium contaminated sites also have reported patterns of cross‐site microbial heterogeneity and association with geochemical parameters including: Oak Ridge, TN where it has been shown that 16S rRNA data is able to distinguish between U contaminated and uncontaminated sites (Smith et al. 2015), and Rifle, CO where it was shown that shorter physical distance differences between microbial communities were ascribed to geochemical or topographical differences between sites (Hug et al. 2015).

The Riverton soil community membership has been shown by Tolar et al. (2020), to remain stable over seasonal hydrological and geochemical redox conditions, whilst varying by depth (Tolar et al. 2020). Although membership varied, many locations tested in this study displayed capacity for the same metabolisms (Figures 5, 6, 7), suggesting shared metabolic pathways enabling the community to respond to seasonal changes by altering metabolisms performed, for example, using different terminal electron acceptors (e.g., oxygen, nitrate, Mn, and Fe).

### 4.2 Metabolisms Observed Across the Site and Relationships to Uranium Oxidation State

We also hypothesized that the microbial community reduction–oxidation metabolism potential will vary spatially, with activities of and members with known reductive metabolisms being found closer to the naturally reduced zone by the Little Wind River, and more oxidizing metabolisms found in the FTA region. Our results partially support this hypothesis, for although the microbial community varied by geochemistry (Figure 4), metabolism results generally varied less. It is possible that these microbial members that perform metabolisms with undetected or minimal results did not survive pumping, transport, or test preparations for example, from oxygen toxicity to some S‐reducing microorganisms (Marschall et al. 1993) impacting their ability to grow in laboratory media or were not members of the planktonic community. Others have shown that S‐ and Fe‐ reducers have higher abundances in sediment‐associated communities than the planktonic (Flynn et al. 2013). Furthermore, the well‐known “Great Plate Count Anomaly” (Staley and Konopka 1985) means only certain members will grow outside of their natural environment and display phenotypes of interest. Overall, the Riverton aqueous microbial community displayed equal potential across the site for NR (Figure 5C), MnR (Figure 6C), and SO (Figure 7B) metabolisms, and similar levels for FeR metabolism (Figure 6A). Convincing evidence of direct microbial enzymatic reduction of U(VI) metabolism and AO metabolism was not seen in any sites (Figures 5B and 6E).

**FIGURE 9:**
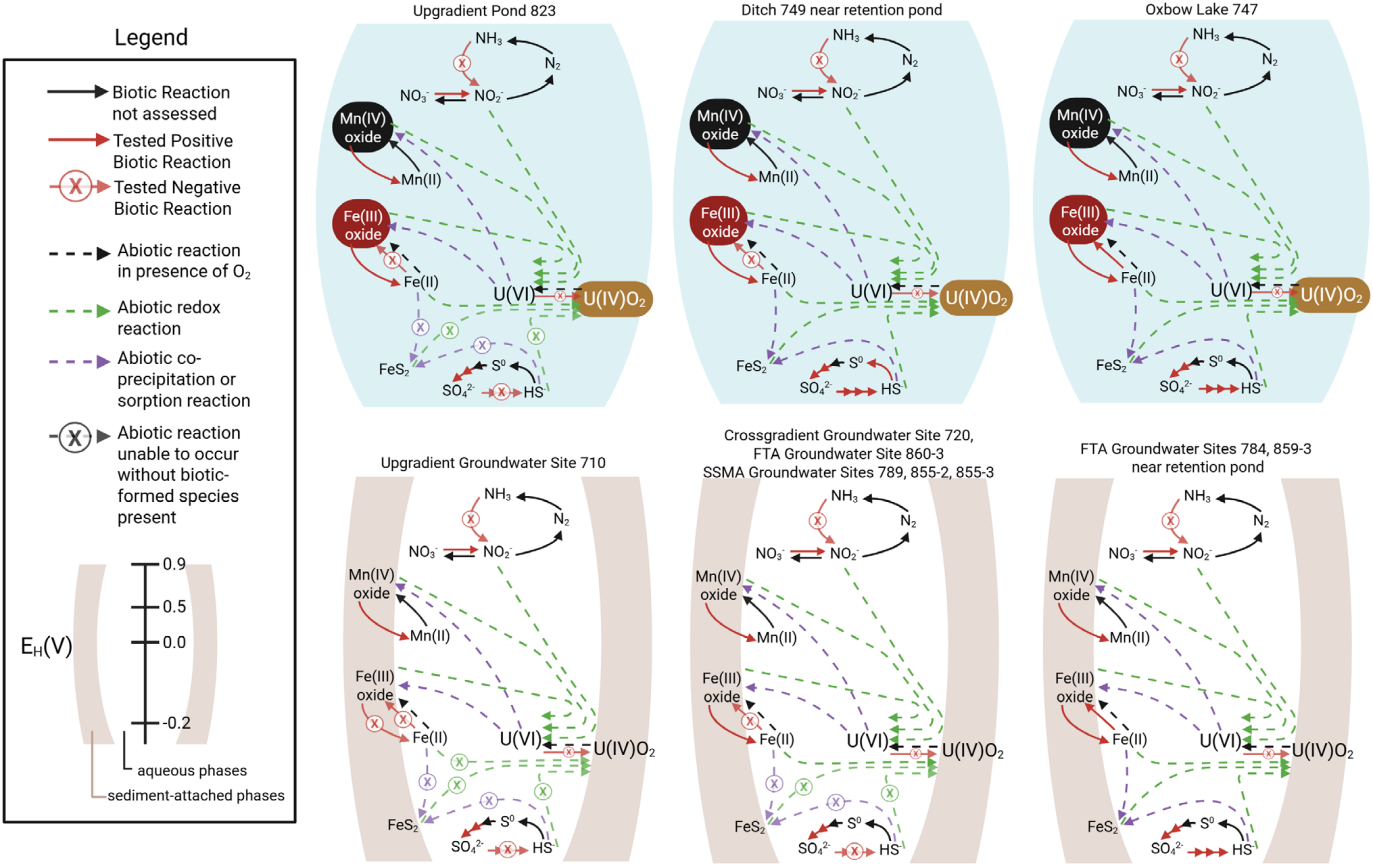
Microbial community metabolic pathway and potential for uranium interaction models of Riverton site regions. Surface water sites are depicted in blue with suspended particles as filled ovals. Groundwater sites are demonstrated as pore‐spaces, with sediment‐associated phases in brown‐regions and generally aqueous phases in the center of each. Biotic reactions are solid lines and abiotic reactions dashed lines. Purple and green dashed lines include secondary abiotic reactions from biotically transformed chemical species.

Riverton sites with a similar set of detected microbial reduction–oxidation metabolisms in this study were grouped, and these reactions along with their relationship with U oxidation or reduction were visualized in Figure 9. Three surface water and three groundwater groups emerged from variabilities in FeR, FeO, and DSR. FTA groundwater sites 784 and 859–3 nearest the retention pond were grouped based on FeR, DSR, and FeO capability. Groundwater cross‐gradient site 720, FTA site 860–3, and all SSMA sites were grouped based on FeR capability and the inability of DSR and FeO metabolisms. This does not support the hypothesis of more reductive metabolisms occurring nearer to the naturally reduced zone by the Little Wind River, for although the oxbow lake near the river demonstrated DSR, the SSMA groundwater sites did not. Rather, the groundwater sites in the FTA nearest the retention pond demonstrated this metabolism. The hypothesis that oxidative metabolisms being found in the FTA is mostly supported. SO was found throughout the aquifer and FeO was found at FTA sites 784 and 859–3; however, AO was not found at any site of the aquifer.

Nitrate reduction metabolism is of interest because nitrate is a frequent co‐contaminant with U (Riley et al. 1992), and microbial nitrate reduction to nitrite (Figure 5A, reactions 1 and 4) can indirectly oxidize and mobilize U(IV) (Figure 9) (Westrop et al. 2023). Depletion of nitrate was seen in all results (Figure 5C) alongside gas production. Although nitrate may have been assimilated as well, ammonium provided in the media inhibits nitrate assimilation (Van'T Riet et al. 1968; McCarty and Bremner 1992) and serves as a more direct source of N for assimilation (Stevens et al. 2024). Cross‐site potential for nitrate reduction matched results from other sites; nitrogen metabolisms in groundwater communities have high redundancy although the major members present vary (Mosley et al. 2022; Anantharaman et al. 2016). Denitrification metabolism evaluation at Oak Ridge, Tennessee by Cardenas et al. (2008), also found that this metabolism was stimulated with the addition of a carbon source. Another study using sediments from Shiprock, New Mexico showed microorganisms capable of multiple anaerobic metabolisms preferentially reducing nitrate prior to Fe(III) and U(VI) reduction and that nitrate can act as an inhibitor for these metal reductions (Finneran et al. 2002). At the Riverton site, nitrogen as nitrate and nitrite is at relatively low concentrations in the groundwater; the last reported measurements for all groundwater values were between 0.01–2.5 mg/L and most values within the U plume were less than 0.4 mg/L (2015 measurement) (Geospatial Environmental Mapping System (Gems), n.d.). This is far below other DOE managed sites where nitrate and nitrite have been more recently measured, for example, Rifle CO Disposal site with levels up to 26.2 mg/L (2024 measurement) (Geospatial Environmental Mapping System (Gems), n.d.) and Tuba City AZ Disposal site with levels up to 725 mg/L (2025 measurement) (Geospatial Environmental Mapping System (Gems), n.d.). Therefore, although nitrate reduction capacity was ubiquitous in surface and groundwaters in Riverton, nitrate reduction as a source of nitrite to abiotically oxidize reduced uranium is likely not an important biogeochemical pathway at this site.

Another potential source of uranium‐interacting nitrite is ammonia/um oxidation which produces nitrite as an intermediate (Figure 5A, reaction 5). Metabolism results did not identify AO occurring in our tests (Figures 5B and S1A). Other studies found ammonium oxidation gene *amoA* present in Riverton aquifer microbial genomes above, at, and below the water table in sediment (Reji et al. 2022) and in similar environments (Cardarelli et al. 2020). This may indicate a difference between potential genomic ability and activity displayed under conditions tested. Due to the lack of evidence of directly measured AO metabolism in the present study, the microbial models in Figure 9 are not shown with AO metabolism.

Biogenic and chemically‐precipitated manganese oxides can oxidize reduced U(IV) and bind U(VI) (Wang et al. 2013). Mn reduction assay showed complete depletion of Mn(IV) in all sites (Figures 6C and 9). Furthermore, aqueous Mn concentrations were shown in Table 2 and Mn(II) species were predicted with PHREEQC (Table S4) across all sites tested, suggesting that active Mn reduction occurs at the site. Mn oxidation tends to occur biotically as it is thermodynamically favourable in oxidizing conditions, but requires a high activation energy and is thereby kinetically controlled by microbial activity (Nealson 2006). Overall, Mn was shown as mostly reduced and microbial communities as contributors to Mn(II) generation and thereby U mobility.

Our results suggest that microbial iron cycling metabolisms occur at the Riverton site and previous literature details many routes of Fe interaction with U. Fe reduction occurred at all sites except upgradient site 710 (Figure 9), which is outside of the groundwater plume. Aqueous Fe was detected at all plume locations (Table 2), suggesting that Fe reduction is actively occurring at the site. Biotically reduced Fe(II) can contribute to partial abiotic reduction of U(VI) to U(IV) (O'Loughlin et al. 2010), but this typically occurs once the aquifer has reached sulfate‐reducing conditions where biogenic FeS species can act upon U(VI) (Veeramani et al. 2013; Janot et al. 2024; Pidchenko et al. 2023). Furthermore, the U species are mostly Ca‐uranyl‐carbonates (Table S4). These U species have lower redox potentials than Fe(III) (Singh et al. 2013) and a decreased reduction capability in the presence of ferrihydrite (Stewart et al. 2011), so it is unlikely for U(VI) reduction to occur prior to Fe(III) reduction. Fe(III) oxides can also contribute to uranium oxidation (Ginder‐Vogel et al. 2006). At Riverton, U has been shown to associate with metal oxides including Fe and Al in the former tailings area (Sultana et al. 2024). This matches with other sites which have reports of U(VI) association with iron oxides including ferrihydrite (Rossberg et al. 2009), goethite, and hematite (Smedley and Kinniburgh 2023). If the microbial community is stimulated to reduce Fe, this could lead to a secondary effect of aqueous U(VI) increase from desorption of U from Fe oxides. However, if the aquifer's reduction potential drops further causing more reducing UR or DSR metabolisms to occur, U will become reduced to uraninite or other less mobile U phases. Three sites—the oxbow lake site 747 and FTA near‐retention pond sites 784 and 859–3, were shown as capable of Fe oxidation (Figures 6B and 9). These were mostly the same sites where biotic sulfate reduction occurred in metabolism tests (Figures 7 and 9), however, these reactions are not thermodynamically favourable to be combined/paired (Appelo and Postma 2004) but rather occur in differing conditions.

Microbial sulfur metabolisms have well established reactions that influence uranium speciation and are of interest at the Riverton site due to elevated sulfate concentrations (Table 2) that correlate with microbial membership (Figure 4, discussed in results Section 3.3). Stimulation tests for sulfur‐species oxidation were consistently positive (Figure 7) across the Riverton site. In the context of groundwater, SO is of interest as sulfide oxidizing bacteria can be an important source of organic C in otherwise limited nutrient regions (Heinze et al. 2023). Recently, Chen et al. (2025) showed that SO can be coupled to FeR and occurs at faster rates biotically than the abiotic reaction alone, linking these two cycles biogeochemically, and suggests that other metal metabolisms may be acting together in ways not previously considered.

The major sulfur reduction pathway in groundwater is dissimilatory sulfate reduction (Figure 7A, reaction 5). Veeramani et al. (2013) showed in controlled laboratory conditions biogenic‐formed mackinawite (an Fe‐sulfide species) as capable of subsequent abiotic U(VI) reduction. In field experiments at Rifle, CO, Janot et al. (2024) showed U(VI) reduction as occurring primarily during the transition from Fe(III) reduction to SO_4_^2−^ reduction, attributing this to the multiple electron donors available as Fe(II), S(‐II), and biomass. These secondary effects of S reduction therefore can drive U reduction despite direct biotic U reduction not being detected (Figures 6E and 9). A study by Pidchenko et al. (2023) also identified thermodynamically stable Ca uranyl carbonates as the primary U species for the Swedish Äspö groundwater aquifer site and modelled U(IV) presence as correlated with HS^−^ concentration. Additional studies show U immobilization as correlated with Fe‐sulfide species pyrite presence (Brown et al. 2016; Yu et al. 2026), although the mechanism of Fe(II) versus S(‐II) species reducing U(VI) varies between studies and interpretations (Brown et al. 2016; Descostes et al. 2010) which may be attributed to the species of U present or precise experimental/field conditions. Perzan et al. (2021) showed S reduction being key for redox condition establishment and maintenance alongside fluid residence time as controlled by aquifer permeability via a Riverton site model. As our S reduction results varied across the microbial community, if and how U reduction occurs will vary. Sulfate reduction was detected in the oxbow lake, ditch near the retention pond, and FTA sites 784 and 859–3 nearest to the retention pond (Figures 7 and 9). During a previous sampling in May 2023, measured groundwater oxidation–reduction potential (ORP) values were lowest for the near‐retention pond region of the FTA (−241.9 to −125) compared to higher values nearer 860–3 (−38.3 to +10.1) (internal DOE Legacy Management database). This matches S reduction capability of the microbial community; generally S reduction occurs at lower ORP levels where other electron acceptor abundances are depleted (Schlesinger and Bernhardt 2013). Taken together, microbial metabolism is likely to be impacting U mobility through different mechanisms across the site based on local area variations in geochemistry and microbial community membership.

### 4.3 Microbial Reactions and Relationship With Remediation Efforts

Typical remediation strategies for U remediation rely on bioreduction, where U is reduced either enzymatically by the microbial community or indirectly by biogenically reduced Fe and S products through C inputs (Bargar et al. 2013; Jeong et al. 2023; Wu et al. 2006). The community at the Riverton site had mixed ability to completely reduce U and S (Figures 6, 7, and 9). Furthermore, the aquifer is susceptible to re‐oxidation through flooding‐drying events (Dam et al. 2015). Therefore, it is unlikely that bioremediation relying on U immobilization would work as intended for long‐term stabilization of reduced U.

Oxidative enhanced flushing has been trialled at the site to increase U flushing from the aquifer. Oxidative enhanced flushing would most likely have the highest impact on areas that demonstrated microbial sulfate reduction and therefore U reduction, for example, FTA sites nearest the retention pond (Figure 1). During enhanced oxidative flushing events to the saturated zone of the SSMA, changes to U concentration appeared to follow expected changes based on tracer concentrations of dilution alone (Applied Studies and Technology 2023). As the SSMA planktonic community was not shown capable of direct U reduction (Figure 6) nor DSR metabolism (Figure 7) which could result in reduced U (Figure 9), this suggests that U is likely found in an oxidized state. This is further supported by geochemical modelling (Table S4) showing mainly U(VI) species across the site. This would explain the SSMA result as oxidation would not occur to an already oxidized U species. However, the FTA saturated zone injections did appear to have an oxidation event occur as U levels rose above initial condition levels (Applied Studies and Technology 2023). Interestingly, although FTA sites nearest the retention pond showed feasible DSR metabolism, FTA community 860–3 closest to the location of the injection experiment did not show this metabolism. Sultana et al. (2024) found U associated with Fe‐rich coatings at this site, suggesting U sorption to Fe as an important factor in this region. Aqueous Fe(II) presence (Table 2) and Fe reduction by microbial communities (Figure 6) were observed, so microbial Fe activity if changed would influence associated U conditions. Further studies will be required to understand the mobilization factors responsible for this uranium increase during oxidative enhanced flushing.

## 5 Conclusion

This study of the aqueous phase microbial community membership and stimulable metabolisms across the Riverton, WY former uranium processing site confirmed the hypothesis that the microbial community varies by site geochemistry, especially sulfate, conductance, and pH. Contrastingly, most of the microbial communities displayed capabilities to utilize nearly the same set of transition metals and inorganic molecules as terminal electron acceptors (O_2_, NO_3_^2−^, Mn(IV), Fe(III)), despite having almost completely distinct microorganisms present. This highlights the resilience of Riverton microbial communities in that they are primed to handle the changing redox conditions in this suboxic and variably reduced aquifer. More broadly, it is another observation of how sequencing alone would not account for system phenotypes and why measuring microbial community activity in conditions replicating in situ conditions is crucial for making management and treatment decisions. Moreover, the instances of uncommonly displayed metabolic capacity in our experiments (Fe oxidation, Fe reduction, and S reduction) contradicted our second hypothesis of reducing metabolisms being found primarily in the near‐river reduced zones at Riverton. Although not connected through a biogeochemical cycle, the highest capacity for Fe oxidation and S reduction was found at the same site but not in an expected location based on the redox state of the site. This again points to the ability of the community to respond to fluctuating conditions. When we combined our microbial results with the site geochemistry and U speciation, we were able to provide an explanation for the lack of U enhanced oxidation during oxic injection observations in the SSMA, as S and U reduction were not found at this site. In the FTA at the site of oxic injections, we found that Fe(III) reduction is a key component of U transport as microbial Fe(III) reduction was observed and U has previously been characterized as associated with Fe. As Fe is reduced by microbial activity to a more aqueous form, this could free associated U where S reduction is not observed. The results of this study provided microbial insights into and potential explanations for the documented efficacy of applied uranium remediation strategies at the different locations within the Riverton site, showing that a uniform cross‐site management approach will likely not be effective. Although this study is site‐specific, the findings are applicable to other variably reduced, metal‐contaminated aquifers.

## Author Contributions

**Catherine J. Pettinger:** methodology, data curation, investigation, formal analysis, visualization, software, writing – original draft, writing – review and editing, validation. **Charles J. Paradis:** conceptualization, funding acquisition, project administration, writing – review and editing, resources. **Allondra M. Woods:** investigation, methodology, writing – review and editing. **Erica L.‐W. Majumder:** conceptualization, methodology, supervision, funding acquisition, project administration, resources, writing – original draft, writing – review and editing. **Raymond H. Johnson:** resources, writing – review and editing, funding acquisition, supervision, methodology.

## Funding

This work was supported by the National Science Foundation (2229870).

## Conflicts of Interest

The authors declare no conflicts of interest.

## Supporting information


**Figure S1:** Metabolism test images taken at the 6 week timepoint. In each image, the left tube is the media control. Note, this left‐most tube is the same in all images within a subfigure. The center‐left tube is replicate‐1 of the metabolism test for the labelled site. The center‐right tube is replicate‐2 of the metabolism test for the labelled site. The right tube is the site‐specific sterile control for the metabolism test.

**Table S1:** Riverton groundwater sample collection site locations.
**Table S2:** Reagents used in experiments.
**Table S3:** Site Geochemistry 2015–2019 as PHREEQC Input.
**Table S4:** Predicted uranium distribution of species at five sites from archived 2015–2019 geochemical data (see Table S3).
**Table S5:** ASV taxonomy identification and count data for sites.
**Table S6:** 16S alpha diversity.
**Table S7:** Percent change values of metabolism tests.

## Data Availability

The data that support the findings of this study are openly available in NCBI at https://www.ncbi.nlm.nih.gov/bioproject/PRJNA1365257, reference number BioProject PRJNA1365257.
