# Supplementary material for: Aquifer Microbial Communities Differentially Display Metabolisms Capable of Secondary Effects on Uranium Speciation Across a Former Metal Processing Site
Source: Environ Microbiol. 2026 Sep 8;28(9):e70411. doi: 10.1111/1462-2920.70411 (PMC13554291; doi:10.1111/1462-2920.70411)
Supplement: Supplementary file 1 [file EMI-28-e70411-s001.docx]

**Supplemental Figure 1.** Metabolism Test images taken at the 6 week timepoint. In each image, the left tube is the media control. Note, this left-most tube is the same in all images within a subfigure. The center-left tube is replicate-1 of the metabolism test for the labelled site. The center-right tube is replicate-2 of the metabolism test for the labelled site. The right tube is the site-specific sterile control for the metabolism test.
